# Effect of Seawater Temperature Increase on the Occurrence of Coastal *Vibrio vulnificus* Cases: Korean National Surveillance Data from 2003 to 2016

**DOI:** 10.3390/ijerph18094439

**Published:** 2021-04-22

**Authors:** Jungsook Kim, Byung Chul Chun

**Affiliations:** 1 Korea Disease Control and Prevention Agency, Cheongju 28159, Korea; jskim1002@korea.kr; 2 Department of Public Health, Korea University Graduate School, Seoul 02841, Korea; 3 Department of Preventive Medicine, Korea University College of Medicine, Seoul 02841, Korea

**Keywords:** *Vibrio vulnificus* infection, seawater, temperature, generalized additive models, relative risk

## Abstract

The purpose of this study was to assess the association between seawater temperature and *Vibrio vulnificus* cases in coastal regions of Korea. All *V. vulnificus* cases in coastal regions notified to the Korea Disease Control and Prevention Agency between 2003 and 2016 were included in this work. Data for seawater temperature on the south, west, and east coast during the study period were provided by the Korea Oceanographic Data Center of the National Institute of Fisheries Science. We used a generalized additive model and performed a negative binomial regression analysis. In total, 383 notified cases were analyzed (west coast: 196 cases, south coast: 162, and east coast: 25). The maximum seawater temperature was the most significant predictor of *V. vulnificus* cases on the south and east coasts (relative risk according to the 1 °C increase in seawater temperature (RR) = 1.35 (95% confidence interval (CI): 1.19–1.53) and 1.30 (95% CI: 1.06–1.59), respectively). However, the mean seawater temperature was the most significant predictor for the west coast (RR = 1.34 (95% CI: 1.20–1.51)). These results indicate that continuously monitoring seawater temperature increase in each coastal area is crucial to prevent *V. vulnificus* infections and protect high-risk groups, such as persons with liver disease.

## 1. Introduction

*Vibrio vulnificus* infection is an acute and fatal infectious disease caused by *V. vulnificus*. *V. vulnificus* is a halophilic Gram-negative bacterium that belongs to the *Vibrio* genus of the *Vibrionaceae* family. This pathogen can cause primary septicemia in high-risk populations, such as individuals with chronic liver disease, diabetes, immunodeficiency, iron storage impairment, and end-stage kidney disease, and it has a fatality rate of 50–60% [1,2]. Besides sepsis, potentially fatal wound infections can occur when wounds are exposed to *V. vulnificus*-contaminated warm seawater during recreational and leisure activities. Moreover, one can be infected with this bacterium through the consumption of uncooked or undercooked seafood [1]. The fatality rate of wound infections ranges from 15% [3] to 25% [4]. Three biotypes of *V. vulnificus* cause severe disease in human; biotype 1 is the most common and accounts for the entire illness, including primary sepsis associated with a fatality rate exceeding 50% [1]. *V. vulnificus* infection requires antibiotic treatment and surgical removal of necrotic tissue [1]. *V. vulnificus* infections are commonly observed in several coastal cities in Japan [5,6], Taiwan [7], and the United States [8,9,10,11]. Moreover, there is a clear seasonality, and cases peak during warm months when the water temperature increases.

*V. vulnificus* can be found in warm coastal regions worldwide and in estuarine environments where water temperatures range from 9 to 31 °C [1]. In an ecological study focusing on the eastern estuarine waters of North Carolina in the United States, *V. vulnificus* was isolated only at water temperatures between 15 and 27 °C. Furthermore, its presence was associated with water temperature, dissolved oxygen levels, and levels of other *Vibrio* species [12]. It is known that high water temperatures greatly promote *V. vulnificus* proliferation and its ability to infect humans. Therefore, water temperature is a reliable predictor of pathogenic *V. vulnificus* infection cases [4,13,14,15,16,17,18]. This bacterium is rarely isolated during winter, when the water temperature in the coastal regions of the Gulf of Mexico falls below 20 °C [4,13,14]. However, the bacterium becomes unculturable at low temperatures, leading to very few cases of infection. The lower limit of growth for *V. vulnificus* is known to be a seawater temperature of 13 °C, and below 13 °C, *V. vulnificus* cells enter the viable but nonculturable (VBNC) state [4]. In South Korea, a country with a high prevalence of *V. vulnificus* infection [17], several studies have assessed the seasonality [19] and geographic distribution of the bacterium [20] and developed mathematical models of the effects of global warming on seawater temperature [21]. However, the effect of water temperature on the occurrence of *V. vulnificus* infection has been poorly assessed quantitatively. Thus, this study was conducted to assess the risk of *V. vulnificus* infection depending on coastal water temperature.

## 2. Materials and Methods

### 2.1. V. vulnificus Cases in South Korea

In South Korea, *V. vulnificus* infection was declared as a notifiable infectious disease on August 1 2000, and population surveillance programs were implemented afterward. A total of 761 *V. vulnificus* infection cases were reported to the Korea Disease Control and Prevention Agency (KDCA) by National Infectious Disease Surveillance System (NIDSS) from 2003 to 2016. Epidemiological investigations were conducted in all 761 of these cases. The data for this study were derived from epidemiological investigations conducted during that period and complete epidemiological datasets. Among these cases, 383 were linked to the consumption of raw seafood or exposure to seawater in coastal cities, counties, and districts.

The present study included confirmed and suspected cases of *V. vulnificus* infection (hereinafter referred to as cases). Cases met the case definition criteria of nationally notifiable infectious diseases put forth by the KDCA in 2020. Confirmed cases included people with clinical symptoms of a *V. vulnificus* infection and a confirmatory infection diagnosis according to the examination criteria. Suspected cases included people with clinical symptoms indicative of *V. vulnificus* infection and epidemiological association (seafood consumption, seawater contact and underlying diseases, etc.) who tested negative for the pathogen.

**Figure 1:**
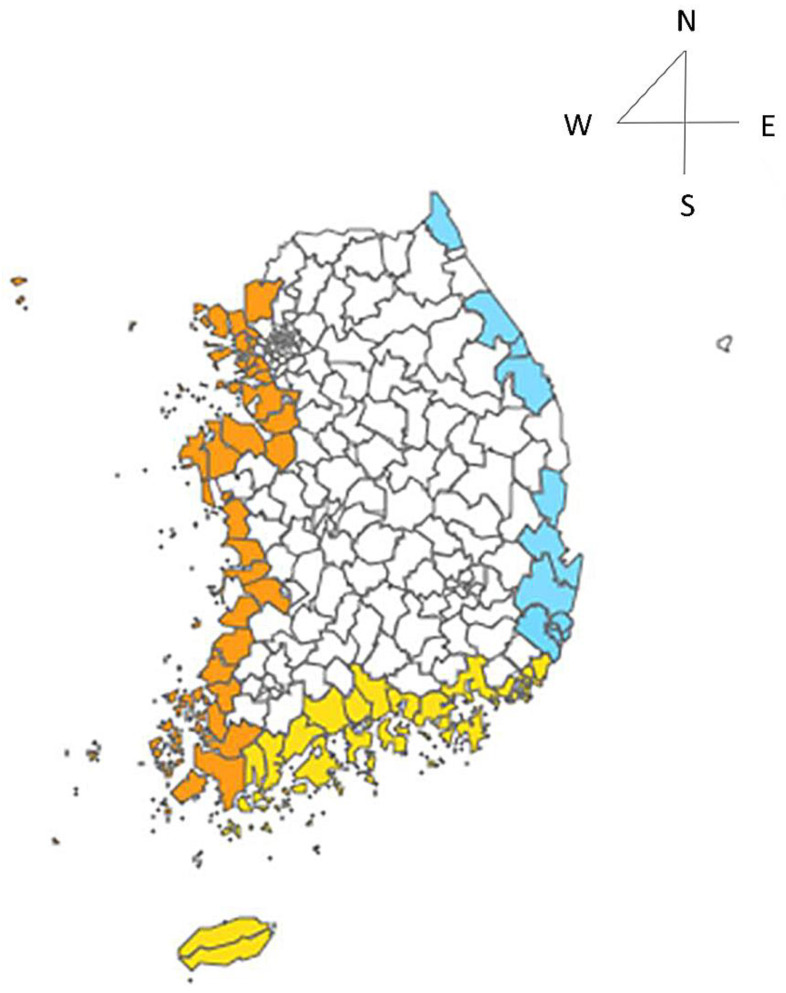
Coastal areas examined in the study.

The subjects of the study were 383 cases residing in the inland or coastal areas of Korea during the study period. They were selected from the 761 cases for which epidemiological investigations were conducted. In the study, the selection criterion for the coastal area was where the 383 cases were infected in cities, counties, and districts adjacent to the sea. Figure 1 shows coastal regions where at least one case occurred. Since Korea is surrounded by three coasts to the west, south and east, Wando and Busan were used as markers to divide the coastal area into 3 sections. Those cities are the stationary coastal seawater temperature observation areas used by the Korea Oceanographic Data Center of the National Institute of Fisheries Science. The coast was divided into three coastal areas: the south coast and the west coast divided at Wando, and the south coast and the east coast divided at Busan.

### 2.2. Seawater Temperatures

To assess the association between seawater temperatures and *V. vulnificus* cases reported between 2003 and 2016, seawater temperature data were obtained from the Korea Oceanographic Data Center. Seawater temperatures were analyzed for each day and coastal region (south, west, and east coasts) to calculate the minimum, average, and maximum monthly temperatures.

Considering that it is more important to examine the duration of high-water temperatures than temperature alone, the number of days within a month on which the seawater temperature exceeded the expected temperature range (18–24 °C) was calculated. The seawater temperature indices were determined through a literature review. The mean temperature at which *V. vulnificus* can proliferate in July (21.2 °C), August (24.1 °C), and September (23.1 °C), as well as at the maximum number of identified cases and the maximum mean temperature in August (24.1 °C) were used in this study.

### 2.3. Statistical Analysis

We performed a descriptive analysis to examine the epidemiological characteristics of confirmed cases at city, county, and district levels. In addition, the absolute water temperature (minimum, mean, and maximum) and the number of days on which the water temperature exceeded the expected temperature range (18–24 °C) were calculated. The link between the number of monthly *V. vulnificus* cases per coastal region and the seawater temperature indices was investigated.

Pearson’s correlation analysis was performed to determine the linear relationship between the monthly number of *V. vulnificus* infections between 2003 and 2016 and each seawater temperature indicator. A generalized additive model (GAM) was applied to predict the nonlinear relationship between the two variables. The monthly number of cases in coastal regions (N) was the dependent variable and the seawater temperature indicators were the independent variables; a smoothing spline function was used. After each coastal model fit, the variable with the lowest Akaike information criterion (AIC) value among the water temperature indicators was selected as a predictor (Equation (1)).

log[E(Yi)] = s (seawater temperature indicators, degrees of freedom: df) + time + seasonality + offset variable(1)

In this equation, E(Yi) refers to the monthly number of *V. vulnificus* cases in coastal regions, and a cubic spline smoothing function was used. The analysis was adjusted for the effector variables time trends (calendar time) and seasonality. The analysis was also adjusted for the population as an offset variable throughout the study period (2003–2016).

Overdispersion (variance greater than the mean) was observed for the dependent variables (monthly number of *V. vulnificus* infection cases). Therefore, negative binomial regression was used to calculate the risk of infection depending on seawater temperature increase. The relative risk (RR) of *V. vulnificus* cases incidence per 1 °C increase in seawater temperature along with 95% confidence intervals (CIs) and the relationship between *V. vulnificus* cases and seawater temperature were analyzed. SAS software version 9.4 (SAS Institute, Cary, NC, USA) and MGCV package in R 3.1.4 software were used for the analysis. The level of significance was set at *p* < 0.05.

The data were publicly available and the institutional review board of Korea University granted exemption for this study (KUIRB-2018-0025-1).

## 3. Results

### 3.1. Research Subjects

**Figure 2:**
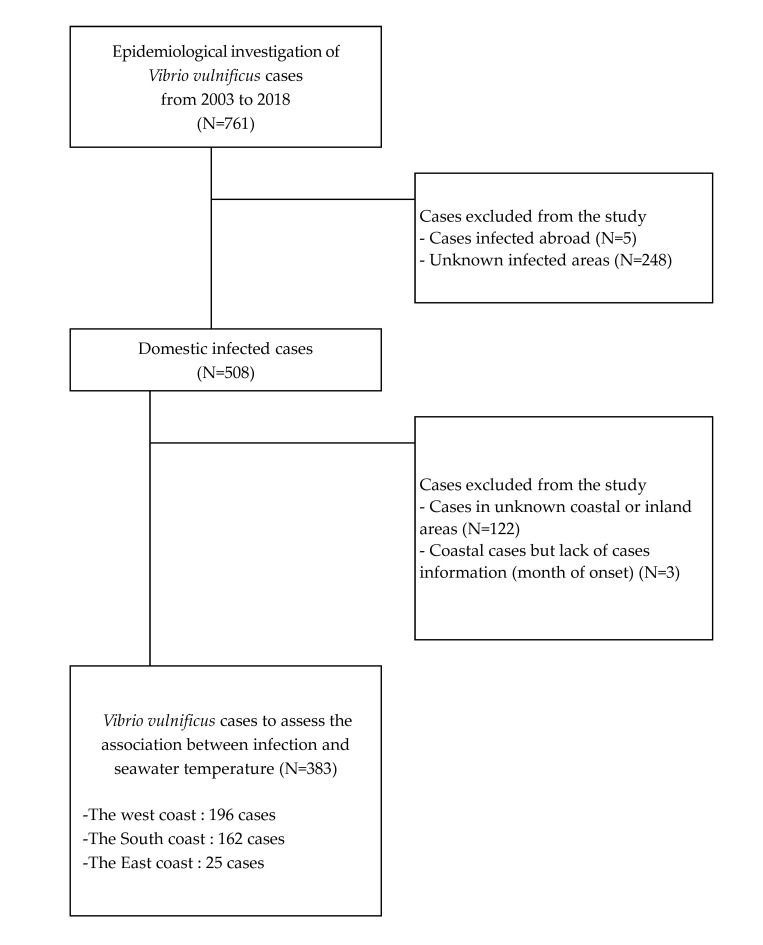
The selection of the study subjects.

A total of 761 *V. vulnificus* cases were reported between 2003 and 2016. Five cases of infections that occurred abroad and 248 with unknown locations at which infection occurred were excluded. Of the 508 remaining cases, 117 registered in the inland area and 8 lacking necessary information (city, county, district, and month of onset) were excluded from the study. Thus, 383 cases in coastal cities, counties, and districts were included in the descriptive epidemiology study and for the analysis of the association between water temperature and *V. vulnificus* infections (Figure 2).

### 3.2. Epidemiological Characteristics of V. vulnificus Cases in Cities, Counties, and Districts Near the Coast

The general and epidemiological characteristics of *V. vulnificus* cases are shown in Table 1. Overall, 329 (85.9%) of the cases involved men, and the mean age of the cases was 58.2 ± 11.2 years. As for occupation, housewife and unemployed were the most common, with 157 (44.7%), and 340 (96.6%) had underlying diseases. According to the lifestyle survey of the cases, 257 (84.5%) had a history of drinking, and 128 (57.4%) had a history of smoking. On the west coast, the rate was the highest, with 196 cases (51.2%).

### 3.3. Annual Indicators Related to Water Temperature and Seawater Temperature

Table 2 summarizes the minimum, mean, and maximum seawater temperatures during the study period.

The mean seawater temperature in Korea between 2003 and 2016 was 15.5 °C. The minimum seawater temperature was the highest in 2016 (12.1 °C), and the maximum seawater temperature was the highest in 2005 (21.0 °C).

### 3.4. Monthly Coastal V. vulnificus Cases in Cities, Counties, and Districts and Seawater Temperature

**Figure 3:**
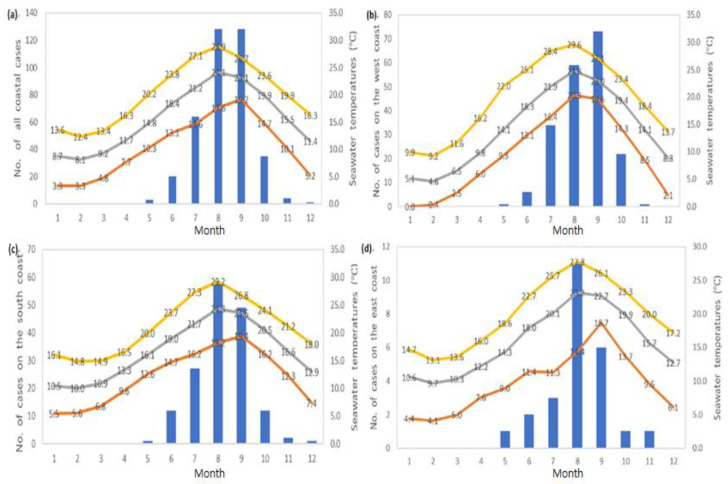
Number of *V. vulnificus* cases and seawater temperatures by month from 2003 to 2016. (**a**) Total cases of all coasts, (**b**) west coast, (**c**) south coast, and (**d**) east coast. Blue bar represents the monthly number of cases, yellow line: maximum seawater temperature, gray line: average seawater temperature, red line: minimum seawater temperature.

*V. vulnificus* cases mostly occurred between July and September of each year (Figure 3). Throughout the 14 years covered by our analysis, the total number of monthly cases was highest in August and September (128 cases per month), followed by July (64 cases). The mean and maximum monthly seawater temperatures of the three Korean coasts were highest in August (Figure 3a). The numbers of *V. vulnificus* infections on the south and east coasts in August were 58 and 11, respectively. However, the number of *V. vulnificus* infections on the west coast was the highest in September, with 73 cases, and the average water temperature was 23.0 °C (Figure 3b). The highest temperatures were 29.2 and 27.8 °C in August on the south and east coast, respectively (Figure 3c,d).

### 3.5. Assessment of the Correlation between Seawater Temperature and V. vulnificus Cases

#### 3.5.1. Correlation Analysis

Pearson’s correlation analysis revealed a positive relationship between seawater temperature and *V. vulnificus* cases and statistically significant correlation coefficients ranging from 0.667 to 0.693 (Table 3).

#### 3.5.2. Results of General Additive Model Analysis

**Figure 4:**
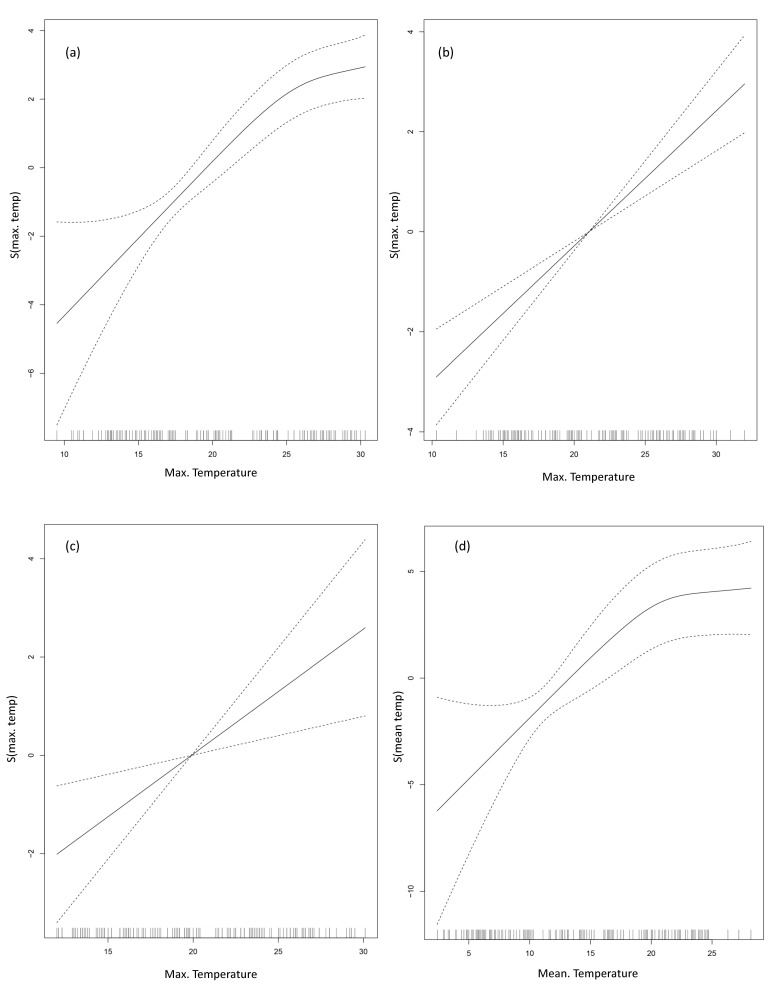
Association between seawater temperature and the incidence of *V. vulnificus* cases on each coast (general additive model analysis). The central solid line represents central estimates, and the dashed line represents 95% confidence intervals. (**a**) All coasts, (**b**) south coast, (**c**) east coast, and (**d**) west coast.

GAM analysis of all coastal, southern coastal, and eastern coastal data showed that the maximum monthly seawater temperature had the lowest AIC value. However, the mean seawater temperature had the lowest AIC value on the west coast (Figure 4).

The adjusted-R^2^ of the water temperature for the incidence of all *V. vulnificus* infection cases was 70%. The adjusted-R^2^ of water temperature for the occurrence of cases was the highest for the west coast at 61%, followed by the south and east coasts at 49% and 22%, respectively.

Table 4 shows the risk of *V. vulnificus* infection cases for every 1 °C increase in seawater temperature on each coast, calculated by selecting the variable with the lowest AIC obtained with a GAM model.

After adjusting for time and seasonality, the maximum seawater temperature was the most reliable predictor of *V. vulnificus* risk on the south and east coasts. The relative risks per 1 °C increase in seawater temperature on the south and east coast were 1.35 (95% CI: 1.19–1.53) and 1.30 (95% CI: 1.06–1.59), respectively. The mean seawater temperature was the most reliable predictor on the west coast and showed a relative risk of 1.34 (95% CI: 1.20–1.51).

## 4. Discussion

The purpose of this study was to assess the association between domestic seawater temperature and *V. vulnificus* cases. *V. vulnificus* cases were most frequently observed in August and September. In August, the mean and maximum seawater temperatures were highest at 24.1 and 28.9 °C, respectively. A positive correlation was observed between each seawater temperature indicator and the number of infection cases. After adjusting for time trend and seasonality, the risk of *V. vulnificus* infection was significantly higher when seawater temperatures were high.

Several *V. vulnificus* cases associated with the consumption of contaminated seafood or exposure to contaminated seawater have been reported in coastal regions worldwide. A total of 4754 cases of *Vibrio* infections were reported by the Centers for Disease Control’s Cholera and Other Vibrio Illness Surveillance (COVIS) system in the United States between 1997 and 2006. Of these, 1210 (25%) cases had developed non-foodborne *Vibrio* infections after being in contact with seawater, and 72% of the *V. vulnificus* infection cases involved residents of Gulf Coast states [10]. In Japan, 94 cases of *V. vulnificus* infection occurred between 1999 and 2000, and 50 (53%) occurred in Kyushu. Interestingly, 43 cases occurred in the coastal regions of the Ariake and Yatsushiro seas, which have several tidal flats [6]. In Taiwan, most of the reported cases (>90%) from 1995 to 2000 involved residents of the southern region of the country [7].

In the present study, *V. vulnificus* cases were frequently observed between July and September in Korea. The number of cases was highest on the west coast, followed by the south and east coasts. This study also shows that the risk of *V. vulnificus* cases increases with increasing seawater temperatures, consistent with findings from previous studies [21,22,23,24,25,26,27,28,29].

The first *V. vulnificus* case in our study was observed in May, when the maximum seawater temperature at the coast was 20.2 °C. In contrast, the maximum seawater temperature from January to April, when cases were not reported, was 12.4–16.3 °C. The cases of *V. vulnificus* infection from 1988 to 2010 reported to the COVIS system in the United States had a distinct seasonal distribution. Infections rarely occurred when the seawater temperature was below 13 °C [30]. It is known that seawater temperature plays a central role in the growth of this bacterium and that infections are rarely reported in winter when the seawater temperature is lower than 15 °C [27].

In this study, positive correlations (r = 0.414–0.805; *p* < 0.001) were observed between the number of *V. vulnificus* infection cases in coastal regions and the nine water temperature indicators. The adjusted-R^2^ of water temperature for the incidence of total *V. vulnificus* infections was 70%. A previous study reported a linear increase in the presence of *V. vulnificus* at seawater temperatures between 11 and 30 °C [24]. In our model, after adjusting for time trend and seasonality, the RR of *V. vulnificus* infection on the south, west, and east coasts according to the 1 °C increase in seawater temperature were 1.35 (95% CI: 1.19–1.53), 1.34 (95% CI: 1.20–1.51), and 1.30 (95% CI: 1.06–1.59), respectively. The estimated exposure–response relationship between *Vibrio* infections and the threshold of 16 °C demonstrated an RR of 1.14 (95% CI: 1.02–1.27) for a two-week lag. The estimated risk was in line with the findings of previous studies [26,29]. For each 1 °C increase in seawater temperature above 20 °C, the probability of the *V. vulnificus* cases increased tenfold [26].

The maximum seawater temperature was the most sensitive index to predict the number of cases on the south and east coasts, while the mean seawater temperature was the most reliable one on the west coast in this study. The highest incidence was observed in August on the south and east coasts when the maximum and mean seawater temperatures were the highest. However, on the west coast, which had the highest infection prevalence among the three coasts, the highest incidence was registered in September, although the seawater temperature was highest in August. Given that the same surveillance and reporting system is used nationwide, delayed reporting cannot explain this discrepancy. However, it has been reported that the abundance of *V. vulnificus* is dependent on the salinity and turbidity of seawater as well as the water temperature [24]. In addition, the increase in the number of *V. vulnificus* cases during hot seasons is not only due to the increased number of bacteria but also to increased exposure to seawater, since more people gather at beaches for leisure activities such as swimming, surfing, and fishing. It has been reported that the amount of *V. vulnificus* in shellfish (oysters) increases when seawater temperature is high, posing greater health risks to people who eat raw oysters [31]. Further studies are needed to explain the discrepancy between high temperatures and the number of cases on the Korean west coast. One of possible explanation for this discrepancy is the characteristics of west seawater. On the west coast, the seawater temperature between June and September is the highest compared to that of the other two coasts, and the turbidity of the seawater is much higher, which may contribute to the growth of bacteria and contamination of the seafood.

The primary limitation of this study is that it was an ecological study that assessed the relationship between seawater temperatures and *V. vulnificus* infection cases in coastal regions. Second, marine environmental factors, such as salinity, turbidity, dissolved oxygen, and suspended particulate matter, which may affect *V. vulnificus* infections, were not considered in this study. Lastly, our work did not consider the number of beach visitors, and behavioral patterns, which may affect the risk of infection in the population during the summer.

The significance of this study is that it is the first to estimate the risk of *V. vulnificus* incidence depending on the increase in seawater temperature in a quantitative manner using national incidence data of over 14 years. Our work also assessed the risk of infection in each of the coastal regions of Korea. Unexpectedly, our findings show that temperature predictors differ between coastal areas and that the sea temperature and the peak case incidence do not coincide on the west coast.

Considering population by coast, the incidence of *V. vulnificus* cases appears different, so it is necessary to further investigate whether liver diseases (cirrhosis, hepatitis, liver cancer, etc.), which are the representative risk diseases for *V. vulnificus* infection, differ by coast.

## 5. Conclusions

This study included 383 registered cases of *V. vulnificus* infection between 2003 and 2016 in coastal regions of Korea. We calculated the risk of *V. vulnificus* infection for every 1 °C increase in seawater temperature on the three coasts. The most reliable infection predictor based on seawater temperature indicators differs between coastal areas. On the west coast, the highest seawater temperature and the peak incidence of cases do not coincide, suggesting that factors other than seawater temperature are also relevant to predict infections. Therefore, it is necessary to consider people’s water activity and eating habits of raw seafood in hot seasons as well as seawater temperature to prevent *V. vulnificus* infections in coastal areas.

## Figures and Tables

**Table 1:** General characteristics of the *V. vulnificus* cases (*N* = 383).

Characteristics	Classification	* N *	(%)
Sex			
	Male	329	(85.9)
	Female	54	(14.1)
Age (years)			
	≤39	13	(3.4)
	40–64	269	(70.2)
	≥65	101	(26.4)
Occupation (*N* = 351)			
	Fishery and fishery product related workers	35	(10.0)
	Agriculture	65	(18.5)
	White-collar workers, professional	18	(5.1)
	Service, distribution industry, self-employed	32	(9.1)
	Blue-collar workers *	44	(12.6)
	Housewife, unemployed	157	(44.7)
Drinking (*N* = 304)			
	Yes	257	(84.5)
	No	7	(2.3)
	Unknown	40	(13.2)
Smoking (*N* = 223)			
	Yes	128	(57.4)
	No	20	(9.0)
	Unknown	75	(33.6)
Underlying diseases (*N* = 352)			
	Yes	340	(96.6)
	No	8	(2.3)
	Unknown	4	(1.1)
* V. vulnificus * cases by year			
	2003	42	(11.0)
	2004	29	(7.6)
	2005	30	(7.8)
	2006	44	(11.5)
	2007	26	(6.8)
	2008	16	(4.2)
	2009	8	(2.1)
	2010	37	(9.7)
	2011	25	(6.5)
	2012	31	(8.1)
	2013	25	(6.5)
	2014	33	(8.6)
	2015	15	(3.9)
	2016	22	(5.7)
Coastal area			
	West coast	196	(51.2)
	South coast	162	(42.3)
	East coast	25	(6.5)
Route of infection			
	Seafood consumption	336	(87.7)
	Seawater exposure	22	(5.7)
	Seafood consumption and seawater exposure	24	(6.3)
	Unknown	1	(0.3)

* Blue-collar workers: construction workers, civil engineers, architects, etc.

**Table 2:** Seawater temperatures and indicators by year from 2003 to 2016.

Year	Min. Seawater Temperature (°C)	Mean Seawater Temperature (°C)	Max. Seawater Temperature (°C)
2003	9.9	15.1	20.1
2004	10.3	15.5	20.6
2005	9.7	15.3	21.0
2006	9.6	15.1	20.2
2007	10.8	15.8	20.5
2008	7.6	15.7	20.9
2009	11.1	16.0	20.3
2010	9.0	15.0	20.1
2011	9.1	14.9	20.0
2012	10.8	15.4	20.1
2013	11.1	15.7	19.6
2014	12.0	16.0	19.8
2015	11.4	15.7	19.4
2016	12.1	16.2	19.5
Mean	10.3	15.5	20.2

**Table 3:** Correlation between seawater temperature indicators and *V. vulnificus* cases.

	Cases	Mean Seawater Temperature (°C)	Maximum Seawater Temperature (°C)	Minimum Seawater Temperature (°C)
Cases	1			
Mean seawater temperature (°C)	0.693 *	1		
Maximum seawater temperature (°C)	0.686 *	0.981 *	1	
Minimum seawater temperature (°C)	0.667 *	0.959 *	0.914 *	1

* Significance at *p* < 0.001.

**Table 4:** Relative risk of *V. vulnificus* cases.

Coast	Seawater Temperature Index	Relative Risk *	95% Confidence Interval	*p*
All coasts	Maximum seawater temperature	1.37	1.24–1.52	0.001
South coast	Maximum seawater temperature	1.35	1.19–1.53	0.001
East coast	Maximum seawater temperature	1.30	1.06–1.59	0.011
West coast	Mean seawater temperature	1.34	1.20–1.51	0.001

* Adjusted for seasonality and time trends.

## Data Availability

The data used this study were extracted from publically available data set. The number of *V. vulnificus* cases has been available from URL: http://www.kdca.go.kr/npt/biz/npp/nppMain.do (accessed on 3 August 2020) [Korean]. The seawater temperature has been available from URL: http://www.nifs.go.kr/kodc/eng/index.kodc (accessed on 3 August 2020).
